# Waugh’s Syndrome in Adulthood: A Case Report of a Rare Combination of Intestinal Malrotation and Ileocolic Intussusception

**DOI:** 10.7759/cureus.111533

**Published:** 2026-06-26

**Authors:** Barbara Freire, Ricardo Lemos, Maria Moreira, Laura Santos, Francisco Sampaio

**Affiliations:** 1 General Surgery, Médio Ave Local Health Unit, Vila Nova de Famalicão, PRT

**Keywords:** adult bowel obstruction, ileo-colic intussusception, intestinal malrotation, intussusception, waugh’s syndrome

## Abstract

Intestinal malrotation is an uncommon congenital anomaly resulting from abnormal rotation and fixation of the midgut during embryologic development. Although typically diagnosed during infancy, presentation in adulthood is rare and frequently associated with nonspecific symptoms. Adult intussusception is also an unusual condition and is commonly related to an underlying pathological lead point. The coexistence of intestinal malrotation and intussusception, known as Waugh’s syndrome, is exceptionally rare in adults.

We report the case of a 76-year-old male presenting with intermittent abdominal pain, increased bowel frequency, and weight loss over two weeks. Physical examination revealed a palpable abdominal mass. Abdominal ultrasonography and contrast-enhanced computed tomography demonstrated ileocolic intussusception associated with intestinal malrotation. Exploratory laparotomy confirmed the diagnosis, revealing abnormal positioning of the bowel and an unreducible ileocolic intussusception. The patient underwent right colectomy with en bloc mesocolic resection and ileocolic anastomosis. Histopathological examination identified an inflammatory fibroid polyp as the lead point. Postoperative recovery was uneventful.

Although rare, the association of intestinal malrotation and adult intussusception should be considered in patients presenting with bowel obstruction and atypical abdominal symptoms. Early recognition and surgical treatment are essential to achieve favorable outcomes.

## Introduction

Adult intestinal malrotation is an uncommon congenital abnormality caused by incomplete rotation and fixation of the midgut during fetal development [1]. Although it is usually identified during infancy because of acute obstructive symptoms, some patients remain asymptomatic until adulthood, when the condition is discovered incidentally during imaging studies or abdominal surgery performed for unrelated pathology [2].

Adult intussusception is also rare and differs significantly from the pediatric form. In contrast to children, where most cases are idiopathic, adult intussusception is frequently associated with an underlying structural lesion acting as a lead point, including benign or malignant neoplasms [3]. Clinical presentation is often nonspecific, which may delay diagnosis.

The coexistence of intestinal malrotation and intussusception, commonly referred to as Waugh’s syndrome, has been extensively described in pediatric populations but remains exceptionally uncommon in adults [4]. Because both entities may present with vague abdominal symptoms or signs of bowel obstruction, imaging studies play a crucial role in establishing the diagnosis and guiding surgical management.

We report the case of a 76-year-old male diagnosed with ileocolic intussusception associated with intestinal malrotation, successfully managed with surgical resection. This case highlights the diagnostic challenges and therapeutic considerations of this rare association in adult patients.

## Case presentation

A 76-year-old man presented to the emergency department with a 15-day history of intermittent abdominal pain associated with increased bowel movements and anorexia. The stools were semisolid, without blood or mucus. He also reported unintentional weight loss during this period. There was no history of fever, nausea, or vomiting.

On physical examination, the abdomen was tender predominantly in the periumbilical and hypogastric regions. A mobile palpable mass was identified in the umbilical area, without signs of peritoneal irritation. Laboratory evaluation revealed leukocytosis (white blood cell count: 17,000/µL, with 90% neutrophils) and elevated inflammatory markers (C-reactive protein: 15 mg/dL). Hemoglobin level was 12 g/dL, while renal function, liver enzymes, coagulation profile, and electrolyte levels were within normal limits.

The patient’s medical history included hypertension and type 2 diabetes mellitus, both adequately controlled with medication. Previous surgical history was notable for a laparoscopic radical prostatectomy. Abdominal ultrasonography revealed a complex bowel mass with characteristic layered concentric rings, producing a “target” appearance on transverse imaging and a “pseudokidney” sign on longitudinal views, findings suggestive of intestinal intussusception (Figure 1). 

**Figure 1:**
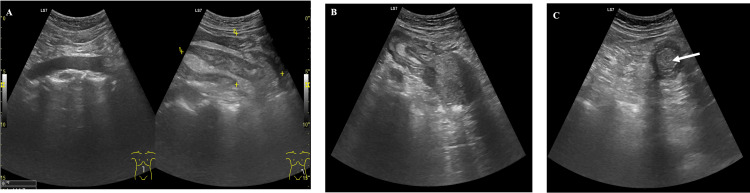
Ultrasound findings of intestinal intussusception A and B – Long-axis sonographic view shows an elongated appearance resulting in a pseudokidney appearance; C – Transverse sonographic view demonstrates a cylindrical mass consisting of an outer hypoechoic ring surrounding a loop of bowel, consistent with intussusception, known as the target sign (arrow).

Contrast-enhanced computed tomography of the abdomen subsequently demonstrated an ileocolic intussusception extending approximately 123 mm, without evidence of bowel ischemia or upstream dilatation (Figure 2). 

**Figure 2:**
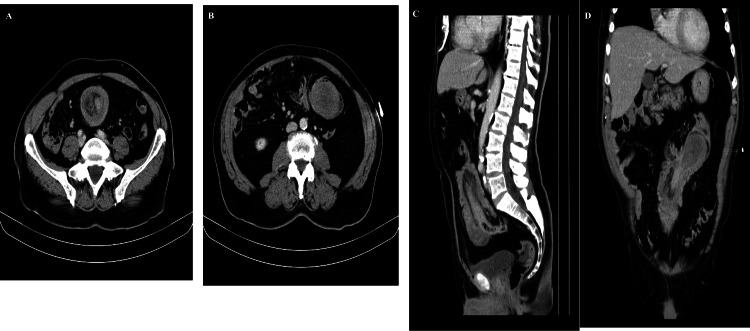
Contrast-enhanced abdominal CT scans demonstrating ileocolic intussusception (A) - Axial CT image showing the characteristic target sign of intussusception. (B) Axial CT image demonstrating an amorphous soft-tissue mass caused by bowel wall edema. Sagittal (C) and coronal (D) CT image showing the ileocolic intussusception and associated lead point.

The patient underwent exploratory laparotomy through a midline incision. Intraoperatively, the small bowel was located predominantly on the right side of the abdomen, while the cecum was abnormally positioned on the left side, where the ileocolic intussusception was identified. The ascending and right colon were unfixed, with the absence of the normal hepatic flexure and persistence of only the splenic flexure, reflecting abnormal intestinal fixation and colonic positioning. These operative findings were consistent with intestinal malrotation. An ileocolic intussusception was identified (Figure 3).

**Figure 3:**
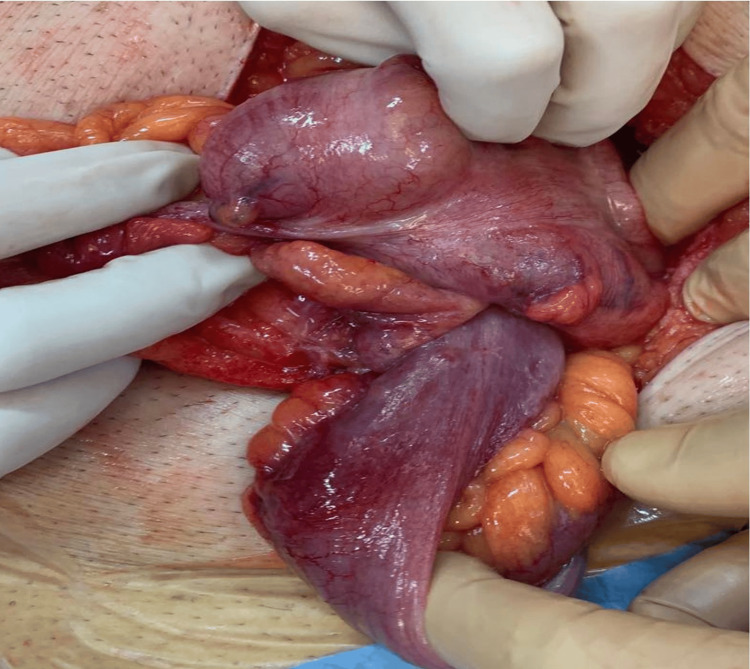
Intraoperative findings demonstrating ileocolic intussusception associated with intestinal malrotation

An attempt at manual reduction was unsuccessful. Given the inability to exclude an underlying malignancy preoperatively, together with the failed reduction, a right colectomy was performed according to oncologic principles, including resection of the associated mesocolon and regional lymphatic drainage, followed by a stapled side-to-side ileocolic anastomosis (Figure 4). The postoperative course was uneventful, and the patient was discharged on postoperative day seven.

**Figure 4:**
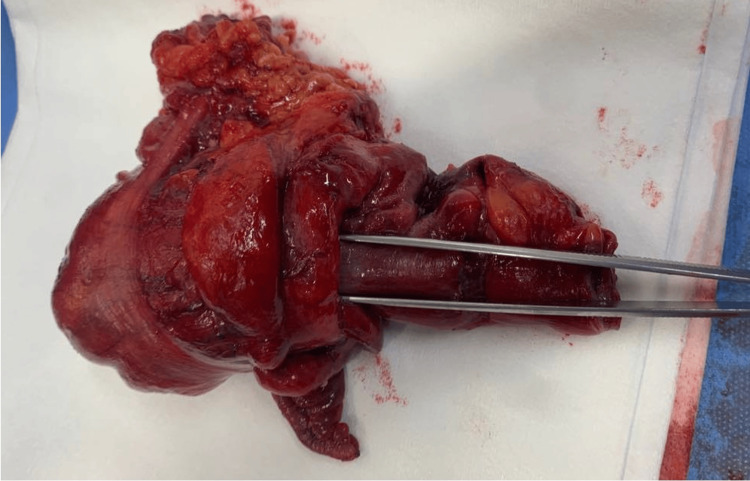
Surgical specimen following right colectomy with en bloc mesocolic resection performed for unreducible ileocolic intussusception

Histopathological examination identified a 5.8 cm inflammatory fibroid polyp located in the terminal ileum adjacent to the ileocecal valve, serving as the lead point for the intussusception. Microscopically, the lesion demonstrated morphological features consistent with an inflammatory fibroid polyp, with a loose edematous stroma and a mild polymorphous inflammatory infiltrate, including rare plasma cells, eosinophils, and numerous mast cells. Extensive surface ulceration and focal hemorrhagic areas were observed, without evidence of dysplasia or malignancy. Immunohistochemical analysis showed the absence of cluster of differentiation (CD) 117 and S100 expression, while CD34 staining highlighted the prominent vascular network. Surgical margins were free of identifiable lesions. The remaining ileal and colonic mucosa showed no significant pathological alterations or dysplastic lesions. Thirteen mesenteric lymph nodes were retrieved and demonstrated only nonspecific reactive changes, with no evidence of malignancy. The patient remained clinically stable during follow-up.

## Discussion

Intestinal malrotation is a congenital abnormality resulting from incomplete rotation and fixation of the midgut during embryologic development [5]. Although it is commonly diagnosed during infancy, adult presentation is uncommon and often identified incidentally during imaging studies, surgery, or autopsy [2]. In adults, symptoms are frequently nonspecific [6], and may include chronic abdominal pain, intermittent obstructive episodes, volvulus, or acute bowel ischemia [7,8].

Radiologic evaluation plays a central role in diagnosis. Contrast-enhanced computed tomography is considered the imaging modality of choice in adults, as it can demonstrate abnormal bowel positioning, inversion of the superior mesenteric vessels, and complications such as volvulus or bowel obstruction [8]. In the present case, CT imaging was essential for identifying the ileocolic intussusception and guiding surgical management. However, intestinal malrotation was not clearly evident on preoperative imaging, likely due to marked anatomical distortion caused by the intussusception, and was ultimately recognized intraoperatively.

Intussusception in adults is rare, accounting for approximately 5% of all intussusception cases and a small proportion of intestinal obstructions [6]. Unlike pediatric intussusception, which is usually idiopathic, most adult cases are associated with an identifiable pathological lead point [9]. Both benign and malignant lesions have been described, including adenomas, inflammatory lesions, Meckel’s diverticulum, metastatic disease, and primary gastrointestinal tumors [9]. In our patient, histopathological examination revealed an inflammatory fibroid polyp acting as the lead point.

Waugh’s syndrome refers to the association between intestinal malrotation and intussusception, classically described in pediatric patients, particularly infants and young children. The diagnosis is established by the coexistence of these two entities, both of which were identified in the present case, with intestinal malrotation recognized intraoperatively and ileocolic intussusception confirmed on imaging and surgical exploration. Although Waugh’s syndrome has been predominantly reported in children, adult cases remain exceptionally rare. Unlike pediatric cases, which are often idiopathic, adult intussusception is more frequently associated with a pathological lead point, including neoplastic or benign lesions. In our patient, an inflammatory fibroid polyp of the terminal ileum served as the lead point, highlighting an important distinction between pediatric and adult presentations of Waugh’s syndrome. The abnormal mobility of the cecum and right colon associated with incomplete intestinal fixation in malrotation has been proposed as a potential predisposing factor for intussusception by facilitating bowel displacement and telescoping. However, in the present case, caution is warranted when inferring causality, as a clear pathological lead point-an inflammatory fibroid polyp of the terminal ileum-was identified. Therefore, intestinal malrotation may have acted as a contributing anatomical factor rather than the sole mechanism underlying intussusception [4,10].

Clinical presentation in adults is often variable and nonspecific, which may contribute to delayed diagnosis. Symptoms commonly include intermittent abdominal pain, nausea, vomiting, altered bowel habits, abdominal distension, or signs of bowel obstruction [6,8]. Physical examination may occasionally reveal a palpable abdominal mass, as observed in our patient [8]. Ultrasonography may provide important diagnostic clues, particularly through the identification of the characteristic “target” and “pseudokidney” signs. [11]. However, CT remains the most sensitive diagnostic modality in adults because it allows detailed evaluation of bowel anatomy, vascular compromise, and potential lead-point lesions, while also supporting surgical planning [12].

Surgical treatment remains the definitive management for adult intussusception. The operative strategy depends on bowel viability, the location of the lesion, and suspicion of malignancy. In colonic intussusception, formal oncologic resection is generally recommended because of the higher incidence of underlying malignant pathology [13]. In the present case, reduction was not feasible intraoperatively, and a right colectomy with en bloc mesocolic resection was therefore performed.

This case highlights the importance of considering uncommon congenital anomalies in the differential diagnosis of adult bowel obstruction. Although rare, the coexistence of intestinal malrotation and intussusception should be recognized as a possible clinical entity in adults, particularly when imaging findings suggest abnormal bowel positioning associated with obstructive pathology.

## Conclusions

Adult intussusception associated with intestinal malrotation is an exceptionally rare clinical entity and may represent a diagnostic challenge because of its nonspecific presentation. Cross-sectional imaging, particularly contrast-enhanced computed tomography, plays a fundamental role in establishing the diagnosis and identifying associated anatomic abnormalities. Early surgical management is essential to prevent complications such as bowel ischemia and obstruction.

This case adds to the limited literature on adult Waugh’s syndrome and highlights the diagnostic challenges of intestinal malrotation in adulthood, particularly when preoperative imaging is obscured by distorted anatomy secondary to intussusception. Characteristic intraoperative findings supported the diagnosis of malrotation in this patient. Furthermore, this case emphasizes the importance of considering congenital intestinal anomalies even in elderly patients presenting with acute or subacute abdominal symptoms, while also reinforcing the need for careful evaluation of a pathological lead point in adult intussusception, as demonstrated by the inflammatory fibroid polyp identified in this case.
